# Electrical resistivity of liquid Fe to 12 GPa: Implications for heat flow in cores of terrestrial bodies

**DOI:** 10.1038/s41598-018-28921-w

**Published:** 2018-07-17

**Authors:** Reynold E. Silber, Richard A. Secco, Wenjun Yong, Joshua A. H. Littleton

**Affiliations:** Department of Earth Sciences, University of Western Ontario, London, Ontario N6A 5B7 Canada

## Abstract

Electrical and thermal transport properties of liquid Fe under high pressure have important implications for the dynamics and thermal evolution of planetary cores and the geodynamo. However, electrical resistivity (*ρ*) and thermal conductivity (*k*) of liquid Fe at high pressure still remain contentious properties. To date, only two experimental investigations of *ρ* of liquid Fe in the pressure region below 7 GPa are reported in literature. Here we report the results of measurements of *ρ* for solid and liquid Fe (inversely proportional to *k* through the Wiedemann-Franz law) at pressures from 3 to 12 GPa, using a large multi-anvil press. We show that *ρ* of liquid Fe decreases as a function of pressure up to the δ-γ-liquid triple point at ~5.2 GPa, and subsequently remains invariant from 6 to 12 GPa, which is consistent with an earlier study on liquid Ni. Our results demonstrate an important effect of solid phase on the structure and properties of liquid Fe. Our values of *ρ* for solid and liquid Fe are used to calculate *k* in Mercury’s solid inner core and along the adiabat in the liquid outer cores of Moon, Ganymede, Mercury and Mars. Our robust values of thermal conductivity place the focus on uncertainties in thermal expansion as the cause of variation in values of core conducted heat. Except for Mercury, our adiabatic heat flux values in these terrestrial cores validate the use of similar values used in several previous studies. Our high values of core adiabatic heat flux in Mercury would provide a stabilizing effect on, and lead to an increase in thickness of, the thermally stratified layer at the top of the core.

## Introduction

Electrical and thermal transport properties of liquid iron (Fe) control the amount of heat conducted through the outer core (OC) to the core-mantle boundary (CMB)^1–7^ in terrestrial planets. Experimental evaluation of electrical resistivity (*ρ*) and thermal conductivity (*k*) of liquid Fe under high pressures (P) is very challenging^4,8^, and remains one of the key aspects in rigorously constraining the thermal, spatial and temporal evolution of terrestrial cores and dynamos^9^. Despite recent advances in high pressure experimental techniques, thus far only two experimental investigations of *ρ* of liquid Fe in the lower P region below 7 GPa are reported in literature^10,11^.

Recent theoretical^2,7^ and experimental^3,4^ studies present a revised set of low *ρ* values and corresponding *k* values for the Earth’s liquid outer core that are up to 4 times higher than previously accepted^1^. Such high values of *k* for the outer core, in the range 90–130 Wm^−1^K^−1^, directly affect estimates of the age of the inner core^9^, as well as the energy budget available to power and maintain the geodynamo^9^ for the past 4.2 Ga^12^. However, in contrast to the low values of *ρ* and high values of *k* in the Earth’s liquid core, the first direct experimental measurements^5^ of *k* of Fe showed *k* to be in line with previously accepted values^1^. New diamond anvil cell (DAC) experimental data^6,13–15^ place constraints on the *k* of lower mantle minerals, which subsequently limits the magnitude of heat flux through the CMB. These experimental results are complemented by numerical studies^16,17^ of Fe at core conditions which demonstrate the important role of electron-electron scattering and spin disorder. The absence of a consensus on *k* and uncertainty in ohmic losses in the core necessitate a consideration of alternative sources of energy required to generate the Earth’s magnetic field throughout history^18^.

It was recently postulated on the basis of theoretical reasoning that *ρ* may be invariant for pure simple liquid metals, but not for liquid transition metals, along their respective melting curves^1^. However, new experimental work demonstrated that transition metals, Ni and Co have invariant *ρ* along the melting boundary^19,20^. This study is motivated by the possibility that liquid Fe may exhibit the same melting boundary behavior of *ρ* as Ni and Co, and enable better insight into the thermal state and dynamics of experimentally reachable conditions of cores, particularly at the boundary between the solid inner and liquid outer cores, of the small terrestrial bodies Mercury, Mars, Moon and Ganymede.

## Results

### Constant resistivity of liquid Fe on the melting boundary above δ-γ-liquid triple point

**Figure 1:**
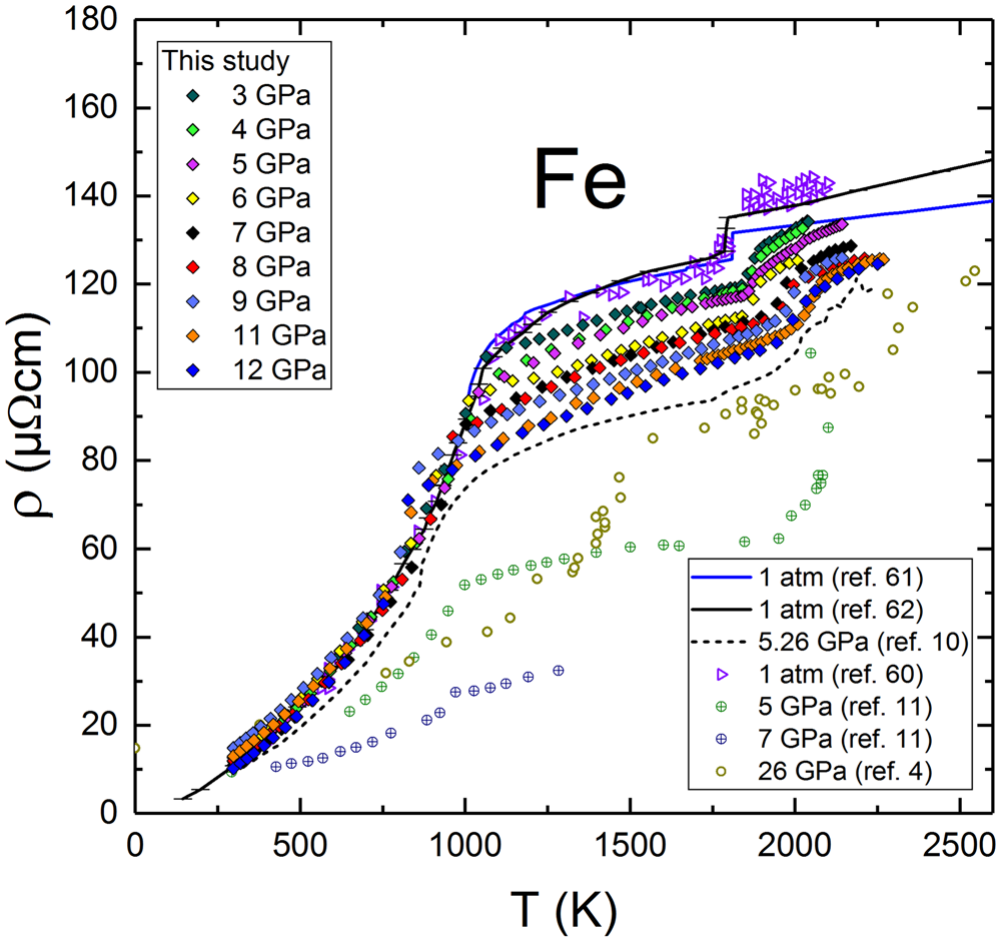
Electrical resistivity of solid and liquid Fe at 3–12 GPa. Our data compared with *ρ* from the earlier results^4,10,11,60–62^, obtained at 1 atm., 5, 5.3, 7 and 26 GPa.

**Figure 2:**
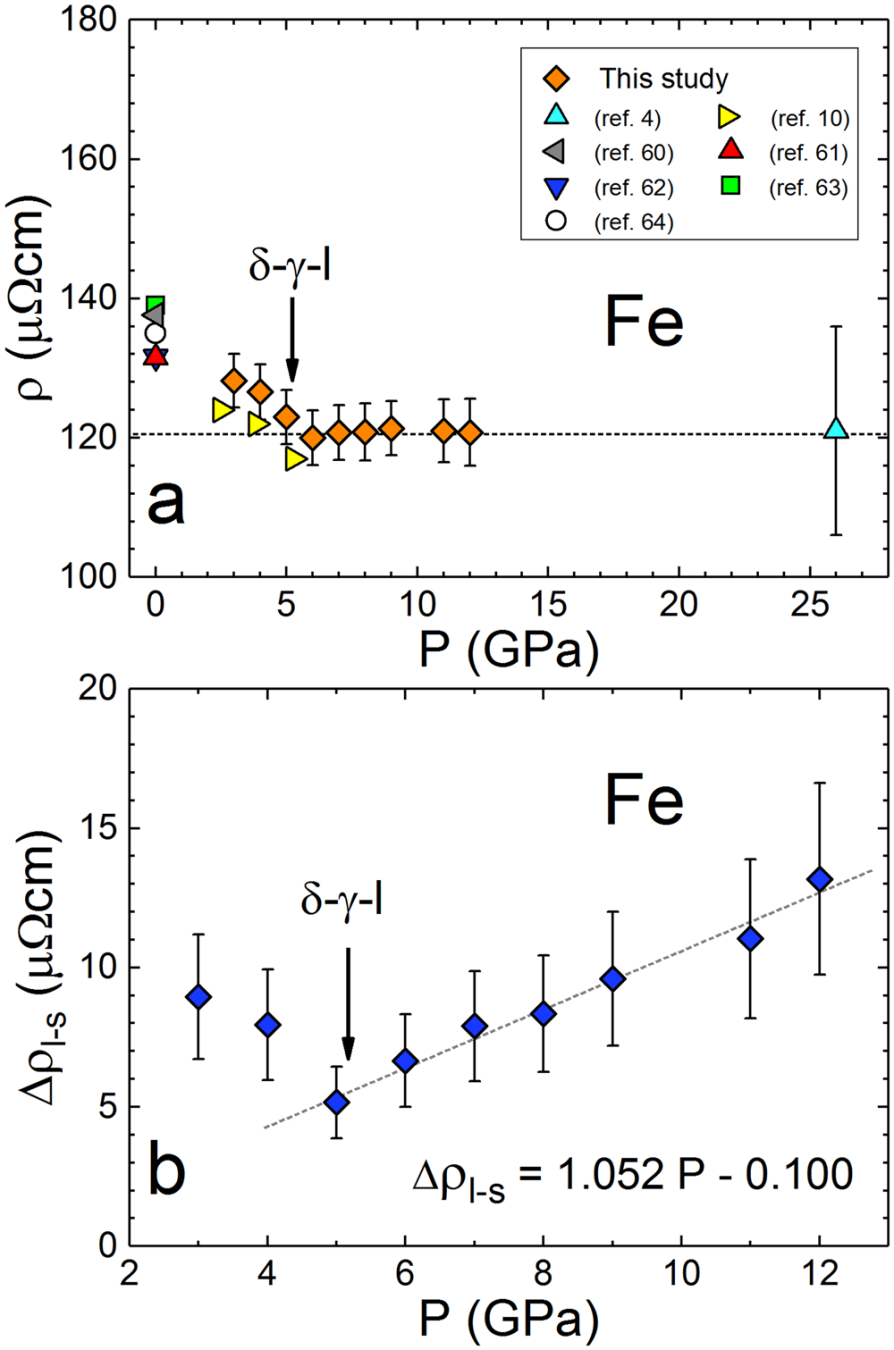
Electrical resisitivity of Fe along the melting boundary and the change in resistivity from solid to liquid. (**a**) *ρ* at the first melt for pressures 3–12 GPa, compared to the earlier results^4,10,60–64^ obtained at 1 atm., 2.5, 3.8, 5.3 and 26 GPa. (**b**) The change in *ρ* on melting demonstrating an abrupt change in trend around 5 GPa. The triple point pressure^32^ is indicated by the arrow.

Here, we report the results of measurements of *ρ* of solid and liquid Fe (99.99% purity) between 3 and 12 GPa (Fig. 1). Our measured *ρ*(P,T) for all experiments are compared to data from atmospheric and other high pressure studies. Overall, the values of *ρ* obtained for solid Fe follow the similar trend observed in measurements at 1 atm and in earlier measurements^10^ up to 7 GPa, but diverge significantly from recent work^11^ for 5 and 7 GPa (Fig. 1). We have not observed the effects of the ε-phase on *ρ* at 12 GPa, nor did we see any deviation from the standard α-phase scattering before the Curie T (T_c_), suggesting that the sample did not enter the ε-phase. The electrical resistivity of α-Fe remains invariant as a function of P up to T_c_ because of strong magnon scattering and a large Fe magnetic moment (2.2μB)^21^. Notably, while T_c_ (1040 K at 1 atm) remains constant up to 1.75 GPa^22^, we observed continuous pressure dependent decrease in T_c_ that approximately follows the α-γ boundary. The electrical resistivity decreases almost linearly above T_c_ as a function of P in the γ-phase, primarily because the long-range order of spin magnetic moments is lost by the effects of temperature, and the electron scattering by magnons becomes reduced compared to temperature-induced phonon scattering, which itself is suppressed by the effects of pressure. Our results demonstrate, within experimental uncertainties, that *ρ* of liquid Fe, along its melting boundary, decreases from 3 to 5 GPa and remains invariant from 6 to 12 GPa. For all experimental P above the δ-γ-liquid triple point at 5.2 GPa, the *ρ* values of liquid Fe at melting remain remarkably constant at ~120 μΩcm up to 12 GPa (Fig. 2a). This value is consistent with recent DAC data^4^ for *ρ* of liquid Fe at 26 GPa.

In comparison with Ni and Co, the interpretation of *ρ* of liquid Fe is more complex due to the polymorphism in solid state before and after the δ-γ-liquid triple point pressure. Such behavior requires additional consideration. Although not previously reported by experimental studies to-date, the abrupt and distinct change in *ρ* of liquid Fe at the δ-γ-liquid triple point is not unexpected. There is also a significant change observed in other properties of liquid Fe at this triple point, such as the temperature coefficient of resistivity^10^, structure factor, compressibility, density^23^ and viscosity^24^.

## Discussion

### Liquid structure, liquid properties and melting boundary

Liquid-liquid phase transitions under high pressure are not uncommon in transition metals^25^. The existence of such a phase transition in liquid Fe around 5 GPa just above the melting boundary, influencing structural changes and many of its properties, has been considered recently^23^. However, we cannot interpret the change in our observed *ρ* of liquid Fe around 5.2 GPa as an equilibrium liquid-liquid phase transition, as such a transition is thermodynamically not plausible at the existing triple point. This leads us to support the premise^10^ that the solid Fe-parent phase has a direct influence on the structure and properties of the liquid in short and medium range order at temperatures very close to the melting boundary. The existence of spin scattering similarities between the liquid and solid Fe-phase^26^ seems to support that. Correspondingly, *in-situ* high pressure X-ray scattering study shows that liquid Fe originating from the γ-parent solid phase remains structurally stable along its melting boundary from 27 to 58 GPa^8^. The aforementioned results impose significant constraints on the thermodynamic and transport properties of liquid Fe^8^. The conclusions of that study imply possible P-independent electron mean free path^19^ along the Fe melting boundary and may corroborate why our observed constant value of *ρ* above 5 GPa for liquid Fe is in line with *ρ*_liquid_ at 26 GPa (ref.^4^) as shown in Fig. 2a. This suggests that such a trend may extend to the ε-γ-liquid triple point.

The structural invariance^8^ of liquid Fe at high P is consistent with a report that the product of thermal expansivity and isothermal bulk modulus (αK_T_) for Fe remains nearly constant at high pressures and temperatures^27^. In the liquid late transition metals under pressure, Peierls/Jahn-Teller distortion (symmetry-breaking rearrangements of atomic structures) and s-d electron promotion^28^ leads to the existence of highly concentrated stable local structures that maximize packing density while lowering electronic binding energy. Although this phenomenon is considered to lower the melting slopes of Fe, Ni and Co, it may also be responsible for maintaining the constant electron mean free path in the liquid at the onset of melt^19^. Indeed, it was found that the packing fraction along the melting curve of Fe remains nearly constant^8^. For Ni, electron-electron scattering was discussed in the context of the Kadowaki-Woods ratio^19^, which compares the temperature dependence of *ρ* to that of heat capacity of a particular metal^29^. The Kadowaki-Woods ratio indicates the presence of strong electron-electron scattering in heavy fermions and weaker scattering in a select group of transition metals^30^, including Fe and Ni. This suggests that the contribution of such scattering to overall *ρ* is not negligible, especially for liquid Fe at high pressure^16,17^.

The jump in *ρ* between the last solid and first liquid (∆*ρ*_l-s_) decreases linearly from 3 to 5 GPa, and then increases at pressures above the 5 GPa δ-γ-liquid triple point (Fig. 2b). We have not measured *ρ* of the ε-phase nor the subsequent ∆*ρ*_l-s_ at corresponding melting points. However, it can be reasonably expected, based on recent results^3^, that *ρ* of the ε-phase saturates at high pressures (above 60 GPa). That would imply that ∆*ρ*_l-s_ from the ε-phase does not increase linearly at high pressures and it likely tapers off. Consequently, given the apparent effects of the δ-γ-liquid triple point on the P-dependence of ∆*ρ*_l-s_, in the absence of experimental results in the liquid above the ε-γ-liquid triple point P, it is not reasonable to speculate on the precise value of *ρ* in Earth’s outer core and at the inner core boundary. However, we do not expect that *ρ* of liquid Fe, originating from a parent ε-phase, would be considerably different from the value of approximately 120 μΩcm, obtained for liquid Fe between 6 and 12 GPa in this study and corroborated by a similar value measured^4^ at 26 GPa. High pressure resistivity measurements on Fe at 300 K on the ε-phase^3^ and demonstration of resistivity saturation at high temperatures^4^ support this. Nevertheless, the increasing magnitude of ∆*ρ*_l-s_ (Fig. 2b) implies that caution needs to be exercised in terms of estimating *ρ* of liquid Fe obtained from solid phase measurement and extrapolated to core conditions.

**Figure 3:**
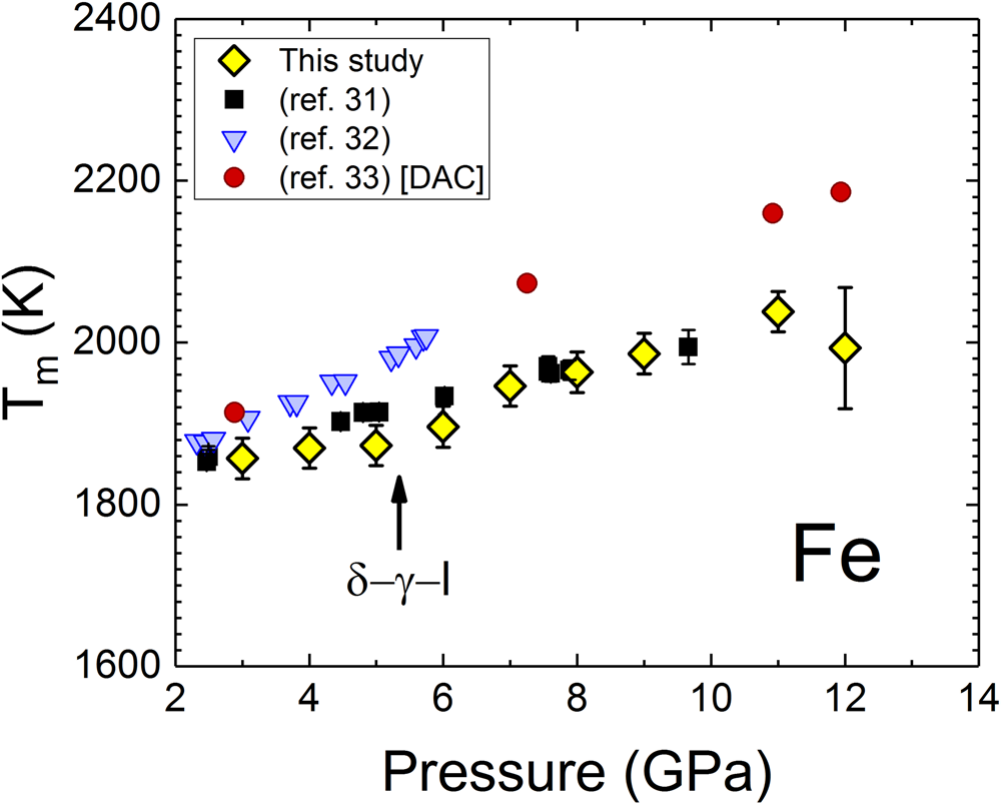
Melting curve of Fe from 3 to 12 GPa. Our melting results, as inferred from resistivity measurements are compared with other studies^31–33^. The triple point pressure^32^ is indicated by the arrow.

We compare our melting curve (T_m_) determined by the jump in *ρ* during the solid-liquid phase transition to available values in the literature (Fig. 3). Our T_m_ values agree well with one study^31^ and are below the values compared to the other two^32,33^. However, there are no other literature data available for the low P melting curve of Fe, thus making the extent of uncertainties due to the choice of experimental set up in previous studies^32,33^ unclear, when compared with our values. It is important to note that the sensitivity of our measurements had sufficiently high resolution to detect the change in slope of the δ-liquid and γ-liquid parts of the melting curve at the δ-γ-liquid triple point, as thermodynamics requires.

### Thermal conductivity and heat flow in the cores of terrestrial bodies

**Figure 4:**
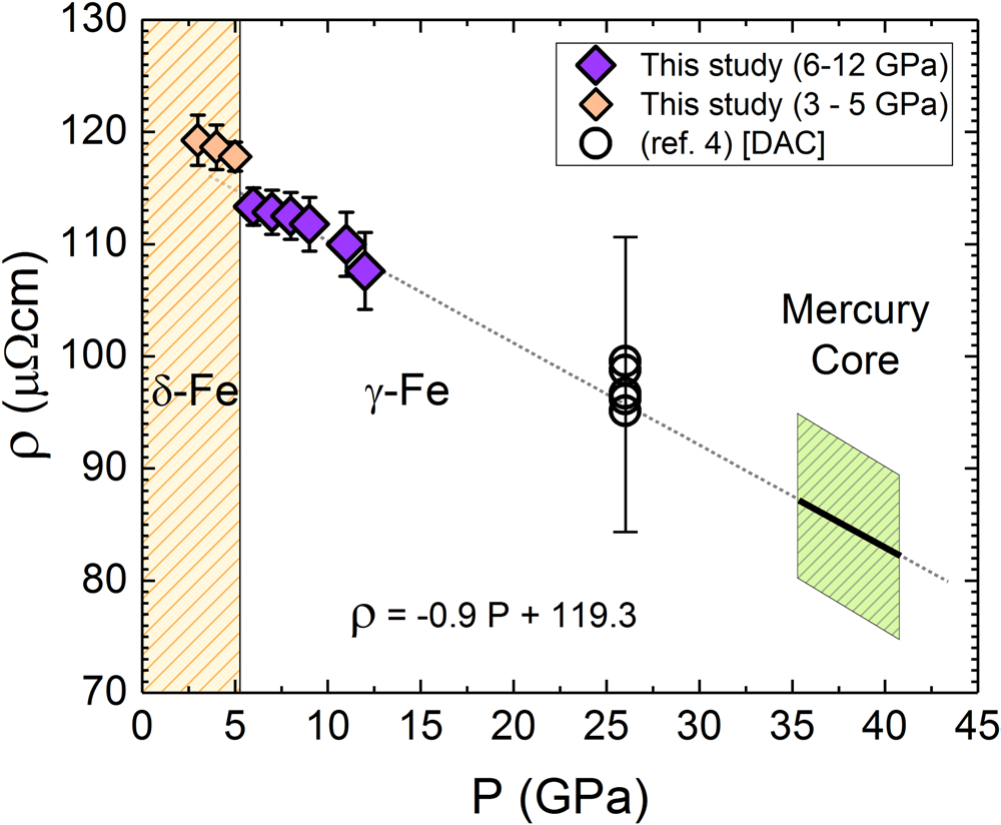
Values of electrical resistivity of γ-Fe just before melting^4^. *ρ* of γ-Fe (6–12 GPa), just before melting, extrapolated to the Mercury core pressure, and compared to *ρ* (with error bars) of γ-Fe before melting^4^ at 26 GPa. Also, we show *ρ* of δ-Fe (3–5 GPa) just before melt (the yellow shaded area on the left). The change in the trend between δ-Fe and γ-Fe as a function of pressure is clearly visible. The green shaded polygonal area on the right represents the uncertainty in *ρ* and P at the center of Mercury’s core.

Our findings have direct implications for constraining electrical and thermal transport properties in the cores of terrestrial bodies made of pure Fe. Mercury has both a solid inner and liquid outer core which generates a weak dynamo^34^. Pressure in the center of Mercury’s solid γ-Fe inner core is about 36 GPa and the temperature is in the range^34^ 2200–2500 K. The pressures at the top of its liquid outer core near the CMB range from approximately 5 to 8 GPa and temperature estimates^35^ are between 1850–2200 K. If we assume a linear decrease of ρ of solid γ-Fe phase just before melting (Fig. 4), we obtain a value for *ρ* of 87 ± 10 μΩcm for an Fe core at the center of the planet. The electronic component of thermal conductivity (*k*_*e*_) is inversely proportional to *ρ* and can be calculated from the Wiedemann-Franz law, ${k}_{e}=\frac{LT}{\rho }$, using for the Lorenz number, *L*, the Sommerfeld value *L*_0_ = 2.44 × 10^−8^ WΩK^-2^, which has been shown to be a reasonably valid approximation^36^ for Fe at Mercury’s core, as well as to much higher P and T^2,37^. For Mercury’s solid core temperatures, we obtain values of thermal conductivity of 62–70 Wm^−1^ K^−1^. At the top of a pure liquid Fe outer core, the value of *k*_*e*_ is calculated to be 41 ± 4 Wm^−1^ K^−1^. The range of thermal conductivity calculated in this study stems from the range of values used for temperatures of Mercury’s CMB. The calculated value of thermal conductivity of 41 ± 4 Wm^−1^ K^−1^, using our measured value of electrical resistivity of liquid Fe at 5 GPa, is in very good agreement with a recently obtained value^36^ of 42.6 Wm^−1^K^−1^ based on measured thermal conductivity of for liquid Fe at 1 atm along with added pressure-dependence of thermal conductivity, as well as a value of 55 ± 8Wm^−1^K^−1^ obtained by first principles calculations^2^ at similar P,T.

We carried out similar calculations for the top of the liquid Fe cores of Moon, Ganymede, and Mars. Thermal conductivity values derived from our electrical resistivity measurements were used with values for thermal expansion (α), gravitational acceleration (*g*), and heat capacity at constant pressure (*C*_*P*_) to calculate the adiabatic heat flow (*q*_*cond*_) on the core side of the CMB of these terrestrial bodies using the following equation:

$${q}_{cond}={k}_{e}{(\frac{dT}{dr})}_{ad}={k}_{e}\frac{\alpha gT}{{C}_{P}}.$$

**Figure 5:**
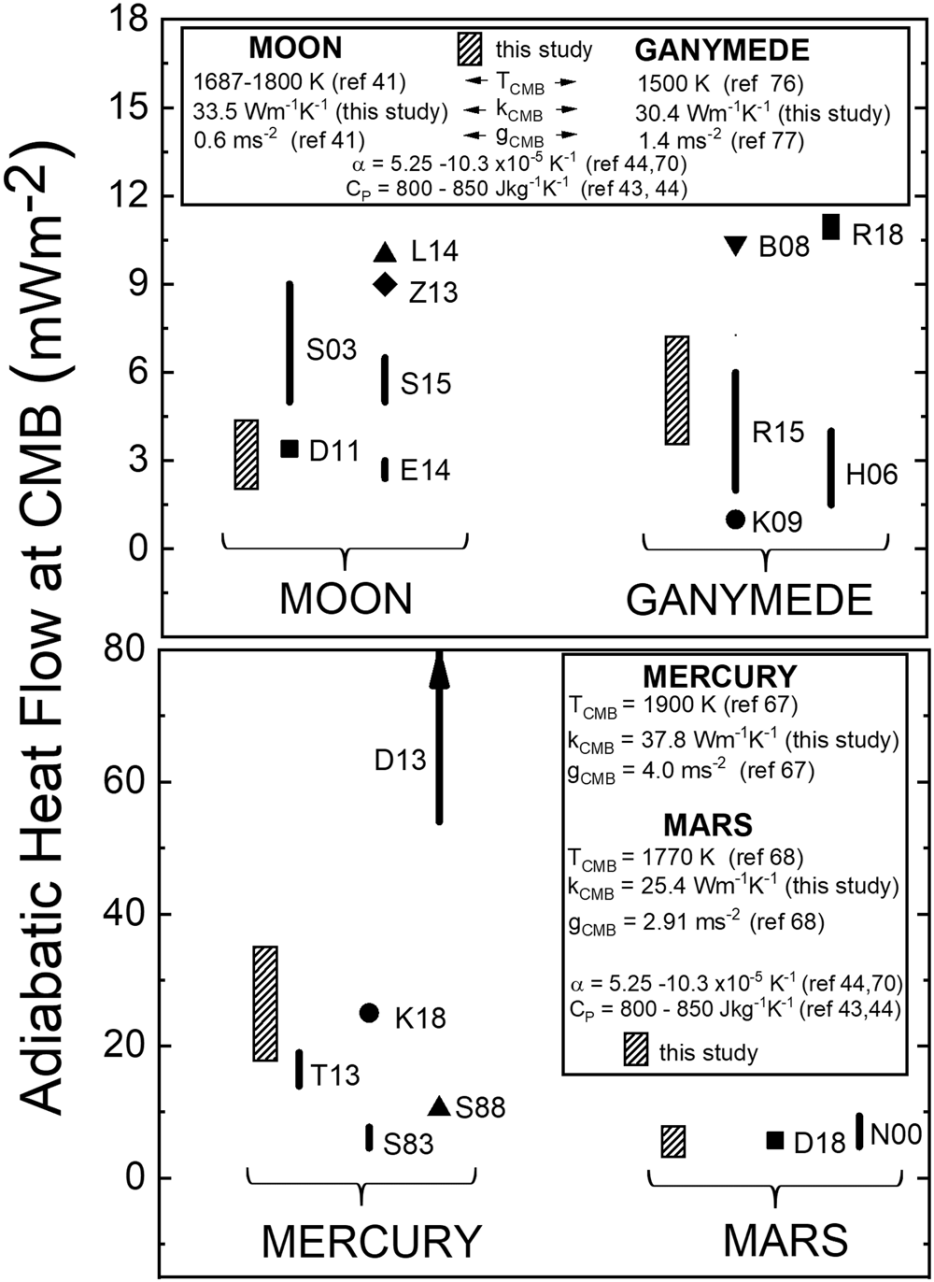
Comparison of values of adiabatic core heat flow at the CMB of Moon, Ganymede, Mercury and Mars. Values obtained in this study (hatched rectangles) were calculated using the parameter values given in the legends. The CMB pressures at which the heat flow values were calculated are: Moon - 4.9 GPa^65^; Ganymede - 5.9 GPa^66^; Mercury - 5.0 GPa^67^; Mars - 23 GPa^68^. References for legend parameters and for heat flow values from other studies^11,38,39,41–47,65–77^ are: B08 - ref.^42^; D11 - ref.^69^; D13 - ref.^11^; D18 - ref.^39^; E14 - ref.^70^; H06 - ref.^71^; K09 - ref.^72^; K18 - ref.^40^; L14 - ref.^45^; N00 - ref.^73^; R15 - ref.^38^; R18 - ref.^43^; S83 - ref.^74^; S88 - ref.^75^; S03 - ref.^44^; S15- ref.^41^; T13 - ref.^47^; Z13 - ref.^46^.

Our ranges of adiabatic heat flow values are compared with several other studies (Fig. 5). The parameter values used in our study are given in the figure legends. The range of values of heat flux conducted along the adiabat is usually attributed to poorly constrained values for thermal conductivity and thermal expansion^38–41^. Our experimentally measured values of electrical resistivity of liquid Fe at high pressures lead to robust thermal conductivity values and this focuses the source of the range of values of adiabatic heat flux on uncertainties in thermal expansion. All other parameters at the CMB of these bodies (*g*, *T*, *C*_*p*_) are known with sufficient accuracy to be responsible for only a few percent variation in the calculated conducted heat flux. Our values of conducted heat flux for Ganymede and Mars are similar in range used in thermal evolution studies. The two high value ranges of conducted heat flux for Ganymede^42,43^ and the three highest values for the Moon^44–46^ are caused almost entirely by the high values of thermal expansion (0.9–1.0 × 10^−4^ K^−1^) used in those studies. However, our range of values for the Moon, which also includes high thermal expansion, is lower than most previous studies of lunar thermal evolution. In part, this is due to the lower thermal conductivity value that we have determined in this study. The comparison for Mercury highlights that the values of conducted heat flow found in our study generally are higher than three previous studies (except for one study^11^, wherein specific details on parameters used in their calculation of conducted heat flow were not provided). Two thermal evolution models^40,47^ used adiabatic heat flux values that fall within our range of values. Most thermal evolution models show the heat flux through Mercury’s CMB to be only a few mWm^-2^ and this is achieved within the first 1 Gyr^47–49^ or even within the first few million years^40^. This means that the heat flow through the CMB is sub-adiabatic which suggests that the outer parts of Mercury’s liquid core are thermally stratified^34^. Our highest values of adiabatic heat flux for Mercury, using the highest values of thermal expansion, would provide a stabilizing effect to the thermally stratified layer owing to the greater departure of the sub-adiabatic temperature profile from the adiabat. This would require greater secular cooling (over longer time period) for the sub-adiabat to intersect the core liquidus. In addition to a stabilizing effect, our high values of core adiabatic heat flux would lead to an increase in thickness of the thermally stratified layer. Both of these effects would slow the growth rate and increase the depth, respectively, of the core region in which Fe snow could develop.

The cores of small planetary bodies are often postulated to include some amount of light alloying elements^50–54^ such as S, Si, O or C. While it has been shown that S, Si, and C in Fe increases ρ for the solid phase at high pressures^55–58^, their effects on *ρ* of the liquid phase, and therefore on *k*, has not been reported. Experimental measurements at 1 atm on Fe-Si compositions with 14–75 wt%Si show that at high Si concentrations, *ρ* in the liquid state is lower than for pure Fe and the temperature dependence of *ρ* in the liquid is negative^59^. This unusual behavior needs to be investigated at high pressure before speculating on the effects on core thermophysical properties and dynamics. Since our results demonstrate an important effect of solid structure on the electrical resistivity of liquid Fe, a direct evaluation of *k*_*e*_ in the Earth’s outer core awaits measurement of *ρ* (or more difficult *k*) for liquid Fe melting from the parent ε-phase.

## Methods

### Instrumentation

We used a large volume 3000 ton multi-anvil press and a 4-wire method, along with a current polarity switch to measure electrical resistivity (*ρ*). The cell design (Fig. S1) and the experimental methods are described in detail elsewhere^19^. The radial geometry of the liquid Fe sample is preserved by using the high density ceramic tube in which a highly polished Fe wire is tightly fitted with tolerances less than 0.01 mm. The only notable difference in this work is the use of a W-foil to contain the Fe melt and preserve the axial geometry of the liquid sample. The advantages of this method^19^ are: (i) liquid containment; (ii) preservation of sample geometry through the full range of P-T experimental conditions; and (iii) delaying the onset of diffusion and sample contamination, such that the *ρ* values collected through rapid data acquisition at the melting temperature are predominantly from the Fe-sample contribution. The experimental approach to evaluate the effects of W diffusion in liquid Fe, and detailed compositional analyses by Electron Microprobe (EMP) data and images, are reported in this section.

### Use of tungsten (W) disc

The most optimal way to measure electrical resistivity of molten materials is to maintain direct contact between the electrodes and the sample. However, such an approach is not possible in the case of liquid transition metals^19,20^ Ni and Co, and even more so in the case of liquid Fe since most electrode materials will easily diffuse into these transition metals. In this work we used type C thermocouples (TC) and diffusion of W and Re from a TC wire junction in direct contact with the molten Fe sample is unavoidable and would inevitably skew readings of both voltage drop and temperature. A modified approach^19^ can be adopted to postpone the effect of diffusion while data are acquired rapidly. This method relies on use of W as an intermediary highly conductive material, located between 4-wire electrode/thermocouple junctions and the sample, enclosed in a high density ceramic tube (CT) (Fig. S1). A W-disc emplaced at these positions is also instrumental in containing molten Fe and maintaining axial geometry. The thickness of W disc is 0.1 mm and a radius of approximately 3 times that of the sample. Considering that electrical resistivity of W is half that of the solid Fe sample at 300 K, the overall contribution to measured *ρ* is less than 1% with the thickness and length utilized in this work. Therefore, the measurement of electrical resistivity of the liquid Fe is minimally affected. Moreover, the choice of W as an intermediary material is influenced by the requirement that its melting temperature is much higher than the melting temperature of the measured Fe sample. By using a W disc as a contact/containment medium, we preserve the chemical (and thermoelectric) integrity of the TC/electrode during the initial rapid set of electrical resistivity measurements that are mainly due to the contribution of the liquid Fe sample. However, such an approach can only be effective if it can be demonstrated that there is little or no immediate diffusion of W throughout the sample, at the onset of melting. To confirm this, we performed EMP analyses on our post-experimental recovered samples that were compressed and heated to temperatures below melting, to the melting temperature, and to temperatures of 50 K and 188 K above melting. The heated experimental cell was immediately quenched after reaching the target temperature. Additionally, we used high density, thick walled, ceramic tubes (Al_2_O_3_) to maintain the lateral geometry of the liquid sample.

### Electron microprobe data

EMP results for samples recovered from heating to high temperatures but not melted showed negligible (<0.4 wt%) W diffusion into solid Fe. The EMP results for samples heated to melting and above are given in Figs S2–S7. Figure S2 shows sample compressed to 3 GPa and heated to the melting point. In the energy dispersive X-ray spectroscopy (EDS) data sets and images shown in Figs S2–S3, it can be seen that at the onset of melting, diffusion of W into the cylindrical ends of the Fe sample takes place. However, the initial set of voltage drop measurements across the liquid sample is primarily due to Fe. The corresponding recovered and sectioned sample is shown in the inset.

In the sample compressed to 7 GPa and heated to 50 K above the melting point (Fig. S4), the evidence of W diffusion into the Fe sample is clearly visible. However, Fe is still dominant at 97.16 at%. Figure S5 shows wavelength dispersive X-ray spectroscopy (WDS) analyses (the annotated values of point analyses correspond to the table values) and the progression of diffusion can be seen. The corresponding optical image of the same recovered sample is shown in the inset of Fig. S4.

In a sample heated to a temperature of 188 K above the melting T, W diffusion in liquid Fe is clearly evident (Figs S6–S7). However, it must be emphasized that diffusion is also sensitive to the amount of time spent in melt. After careful observation, it can be seen in plots of *ρ* (Figs 1 and S9) that the linear positive slope of *ρ* in liquid Fe starts decreasing after the first 3–6 data points in the complete melt. This is interpreted to be caused by the progression of W diffusion in liquid Fe, which starts to lower *ρ*. For all experimental pressures in this work, we observed that the trend line of *ρ* in the liquid changes sharply to a negative slope at about the 16 second mark in melt. We note a rapid decrease in *ρ* after a duration of 16 seconds in the melt, but these data are not shown here. A reasonable interpretation is that after approximately 16 seconds (following the onset of melt), the effect of W in the liquid Fe starts to dominate in the measured electrical resistivity. This leads to rapid decrease of *ρ*, observed in liquid at all pressures in this study. Therefore, in determination of *ρ* along the melting boundary, we only consider those first few *ρ* points in the liquid Fe that exhibit a distinct linear trend.

### Determination of electrical resistivity along the melting boundary

Figure S8 illustrates the procedure of determining *ρ* of Fe along its melting boundary. The procedure consists of fitting a linear trend through data points of *ρ* in the solid and connecting the last *ρ* point in the solid with the points in the liquid using a linear trend through intermediate points between the two phases. The intersection between the linear fit through the first few *ρ* points in liquid (with a clear linear trend) and the line through intermediate points, connecting the solid and liquid phase, is taken to be *ρ* along the melting boundary. It is interpreted that Fe is fully melted at that point so that the measured *ρ* is the resistivity of only the liquid.

### Uncertainties

Representative error bars are shown in Fig. S9 for 3, 6 and 9 GPa of this study. The errors reflect the standard deviation between the absolute value of positive and negative polarity voltage readings during data collection, as well as the uncertainty in length of the sample that arises from volumetric changes at high P and T including melting, and slight changes in shape of the recovered sample. The uncertainty in length in the solid phase is 0.01 mm and in the liquid phase 0.05 mm. Overall, we follow the same methodology in error determination as discussed in detail by Silber *et al*.^19^.

### Sectioned samples geometry

For illustrative purposes, we show images of representative post-experiment samples recovered from a range of pressures that were heated deep into liquid to illustrate the preserved geometry in quenched samples (Fig. S10).

### Data Availability Statement

All data reported in this work are available from the corresponding author (R.A. Secco secco@uwo.ca).

## Electronic supplementary material

Supplementary Information
