# Supplementary material for: Electrical resistivity of liquid Fe to 12 GPa: Implications for heat flow in cores of terrestrial bodies
Source: Sci Rep. 2018 Jul 17;8:10758. doi: 10.1038/s41598-018-28921-w (PMC6050324; doi:10.1038/s41598-018-28921-w)
Supplement: Supplementary file 1 — Supplementary Information [file 41598_2018_28921_MOESM1_ESM.pdf]

# Supplementary Information for:

## Electrical resistivity of liquid Fe to 12 GPa: Implications for heat flow in cores of terrestrial bodies

Reynold E. Silber[1], Richard A. Secco[1]*, Wenjun Yong[1], Joshua A. H. Littleton[1]

[1]Department of Earth Sciences, University of Western Ontario, London, Ontario, Canada, N6A 5B7.

*Correspondence to: R. A. Secco, E-mail: secco@uwo.ca.

## Supplementary Figure Captions

**Fig. S1. The high pressure experimental cell design**.

**Fig. S2. A representative example of EMP-EDS of a sample compressed to 3 GPa** and heated to the melting point used in determination of diffusion of W into Fe at the melting point. The heated experimental cell was immediately quenched after reaching the target temperature. Surface diffusion along the grain boundaries is visible; however overall Fe is 99.42 at%. The yellow outlined rectangular are is the region probed by EDS. Inset: Corresponding image of post-experiment recovered and sectioned sample.

**Fig. S3. Close-up of the region with surface diffusion of W into liquid Fe**. The sample was compressed to 3 GPa and heated to the melting point. The heated experimental cell was immediately quenched after reaching the target temperature. The regions are W (or W/Re TCs) and the dark gray metal in the middle is the Fe sample. The light grey regions at the ends of the sample contain some W.

**Fig. S4. Representative EMP-EDS of a sample compressed to 7 GPa and heated to 50 K over the melting point, and then quenched**. The diffusion of W can be seen throughout the sample which is still dominated on average by Fe (97.16 at%). The upper part of the sample is less contaminated due to the fact that it was on the bottom during the experiment, and diffusion is likely uneven because of the influence of gravity. The rectangular area outlined in yellow is the region

probed by EDS. Inset: The corresponding post-experimentally recovered and sectioned sample. The image in the inset is rotated 180° relative to the EMP image.

**Fig. S5. EMP-WDS of the sample in Fig. S4**. The annotated points on the sample correspond to the point numbers in the table.

**Fig. S6. EMP-EDS of the sample compressed to 3 GPa and heated to 188 K above the melting point**. It is possible to see the formation of dendritic structures at the ends of the sample because the TC wires penetrated the disc and were in touch with the liquid Fe sample, which enabled diffusion of both W and Re into liquid Fe. The rectangular area outlined in yellow is the region probed by EDS. Inset: Magnified dendritic structures in the lower part of the sample.

**Fig. S7. The EMP-WDS scan of individual points on the sample in the Fig. S6**. The points and values in the table correspond to the annotated points throughout the sample.

**Fig. S8. Illustration showing how the ρ along the melting boundary of Fe and Δρ ($\rho_{liquid}$ – $\rho_{solid}$) were determined**.

**Fig. S9. Electrical resistivity curves at 3, 6 and 9 GPa with representative error bars** and combined with available literature values of ρ obtained at atmospheric pressure and at 5, 5.3, 7 and 26 GPa. The simultaneous increases in ρ and T in the melting interval are likely the result of the presence of T-gradients in the cell which are exacerbated by the rapid rate of heating through melting.

**Fig. S10. Representative examples of recovered and sectioned sample cells heated and compressed** to (a) 8 GPa, (b) 9 GPa, (c) 11 GPa and (d) 12 GPa. The maximum temperatures are stated in the figures. Panels (a) – (c) show 18/11 cell and panel (d) shows 14/8 cell. A W-disc was employed throughout.

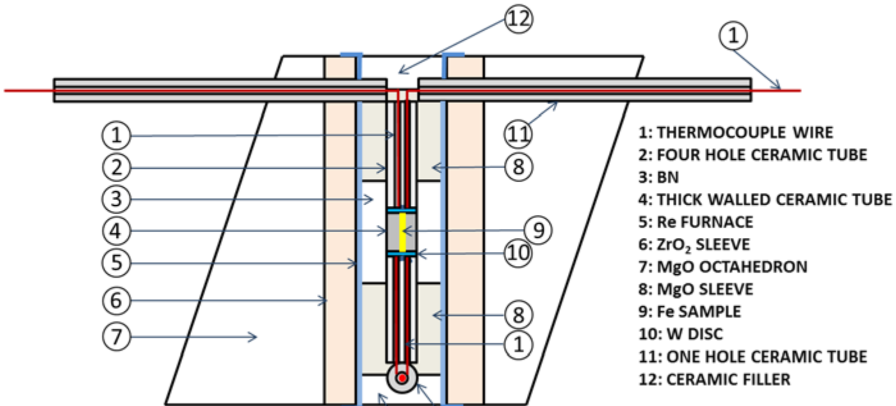
1: THERMOCOUPLE WIRE
2: FOUR HOLE CERAMIC TUBE
3: BN
4: THICK WALLED CERAMIC TUBE
5: Re FURNACE
6: $ZrO_2$ SLEEVE
7: MgO OCTAHEDRON
8: MgO SLEEVE
9: Fe SAMPLE
10: W DISC
11: ONE HOLE CERAMIC TUBE
12: CERAMIC FILLER

Fig S1

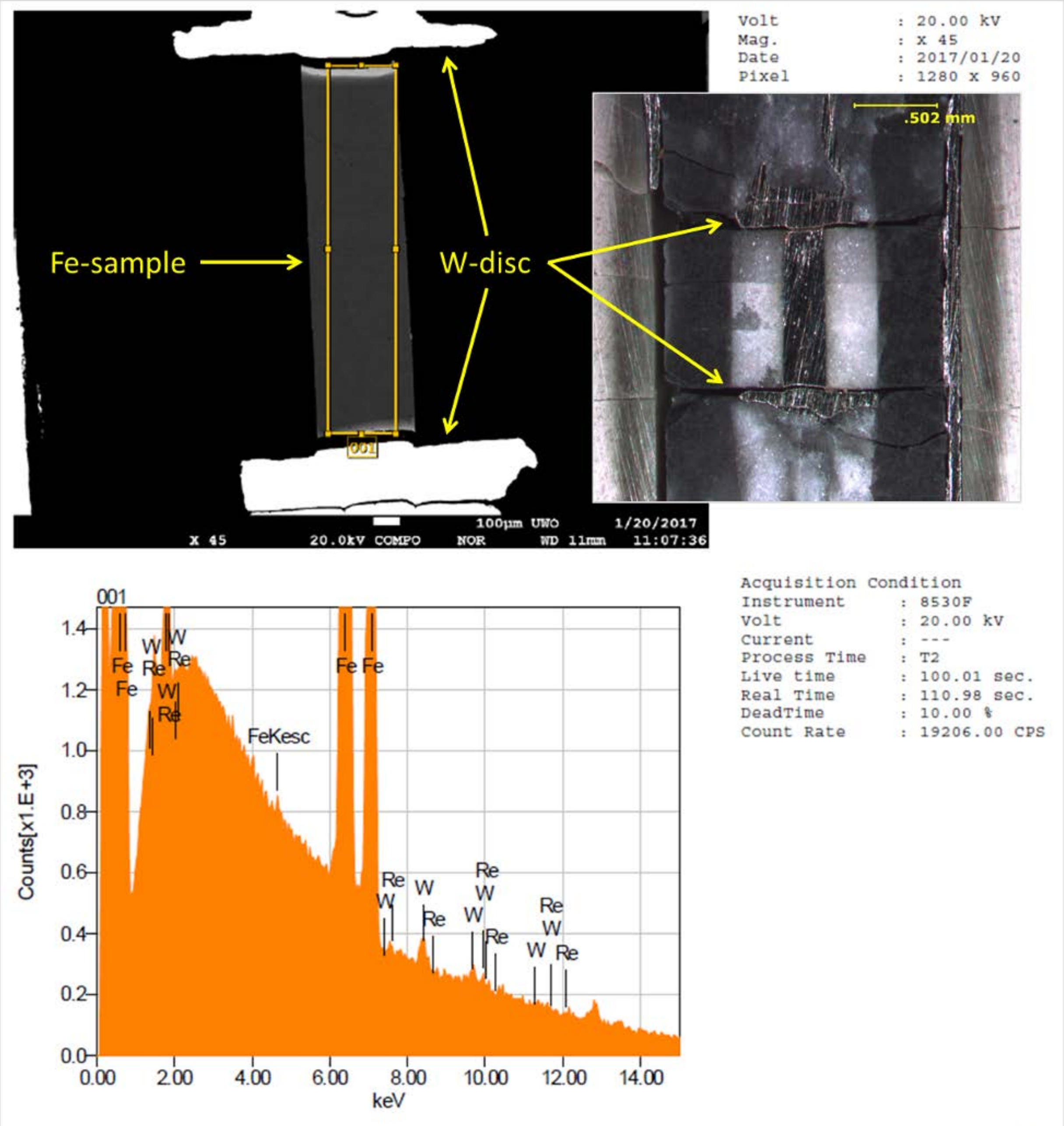


| Formula | mass% | Atom% | Sigma | Net | K ratio | Line |
|---|---|---|---|---|---|---|
| Fe | 98.11 | 99.42 | 0.02 | 24935124 | 51.4994680 | K |
| W* | 1.65 | 0.51 | 0.01 | 338331 | 0.4654793 | M |
| Re* | 0.24 | 0.07 | 0.01 | 52252 | 0.0643842 | M |
| Total | 100.00 | 100.00 | | | | |

Fig S2

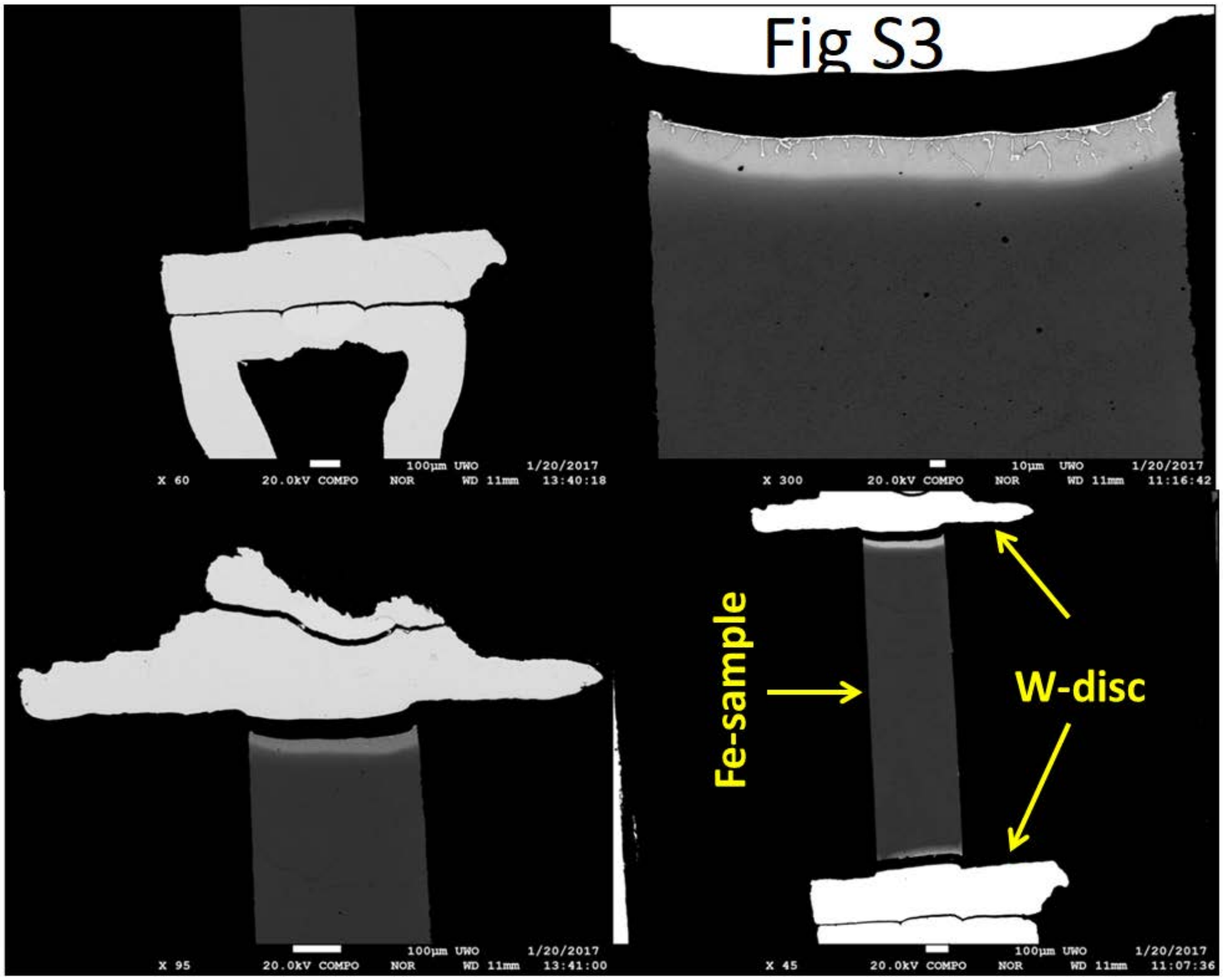
Fig S3
X 60 20.0kV COMPO NOR 100µm UWO WD 11mm 1/20/2017 13:40:18
X 300 20.0kV COMPO NOR 10µm UWO WD 11mm 1/20/2017 11:16:42
X 95 20.0kV COMPO NOR 100µm UWO WD 11mm 1/20/2017 13:41:00
X 45 20.0kV COMPO NOR 100µm UWO WD 11mm 1/20/2017 11:07:36
Fe-sample
W-disc

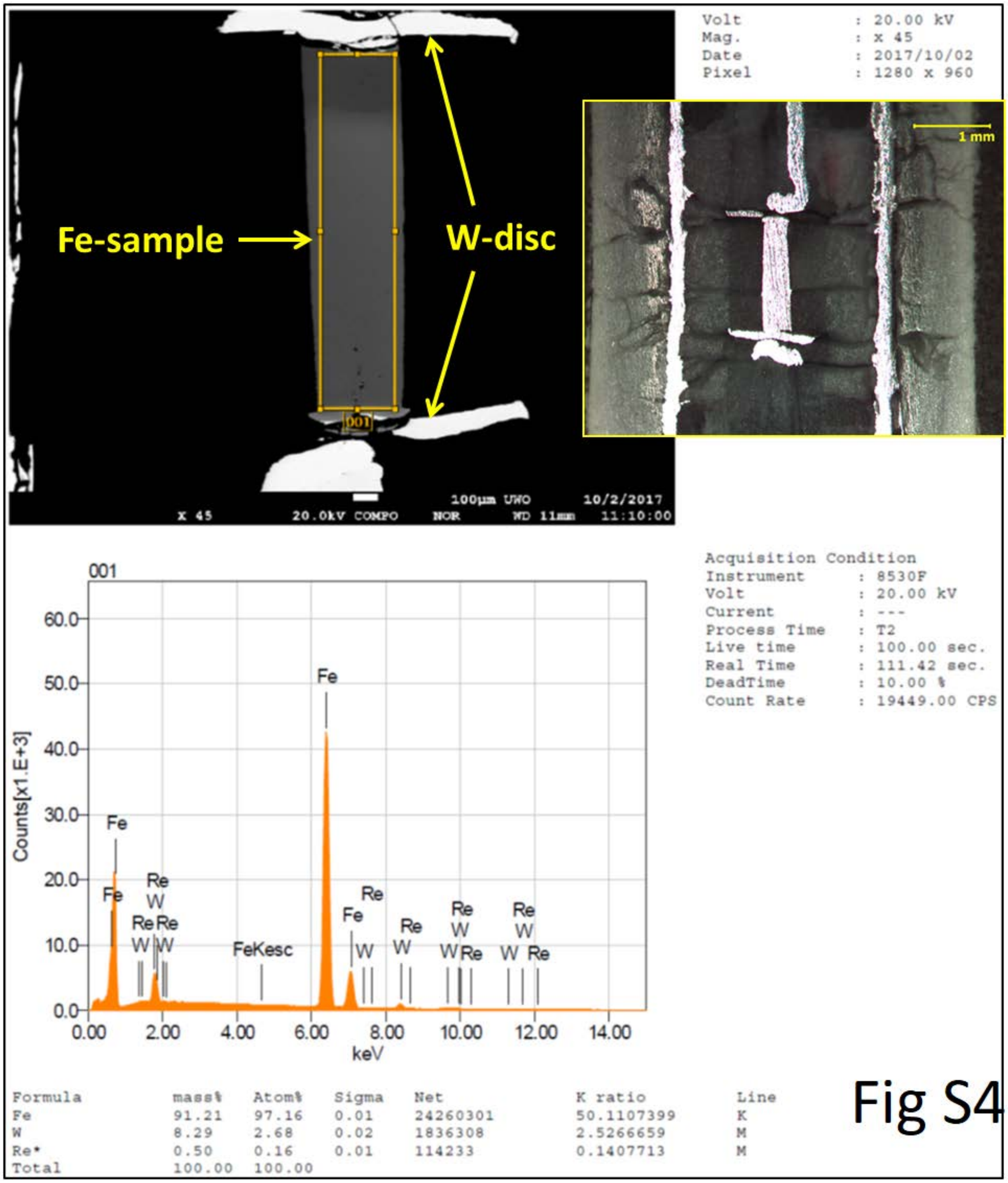
Volt : 20.00 kV
Mag. : x 45
Date : 2017/10/02
Pixel : 1280 x 960
Fe-sample
W-disc
1 mm
100µm UWO 10/2/2017
x 45 20.0kV COMPO NOR WD 11mm 11:10:00
001
Counts[x1.E+3]
keV
Fe
FeKesc
Re
W
Acquisition Condition
Instrument : 8530F
Volt : 20.00 kV
Current : ---
Process Time : T2
Live time : 100.00 sec.
Real Time : 111.42 sec.
DeadTime : 10.00 %
Count Rate : 19449.00 CPS
Formula mass% Atom% Sigma Net K ratio Line
Fe 91.21 97.16 0.01 24260301 50.1107399 K
W 8.29 2.68 0.02 1836308 2.5266659 M
Re* 0.50 0.16 0.01 114233 0.1407713 M
Total 100.00 100.00

Fig S4

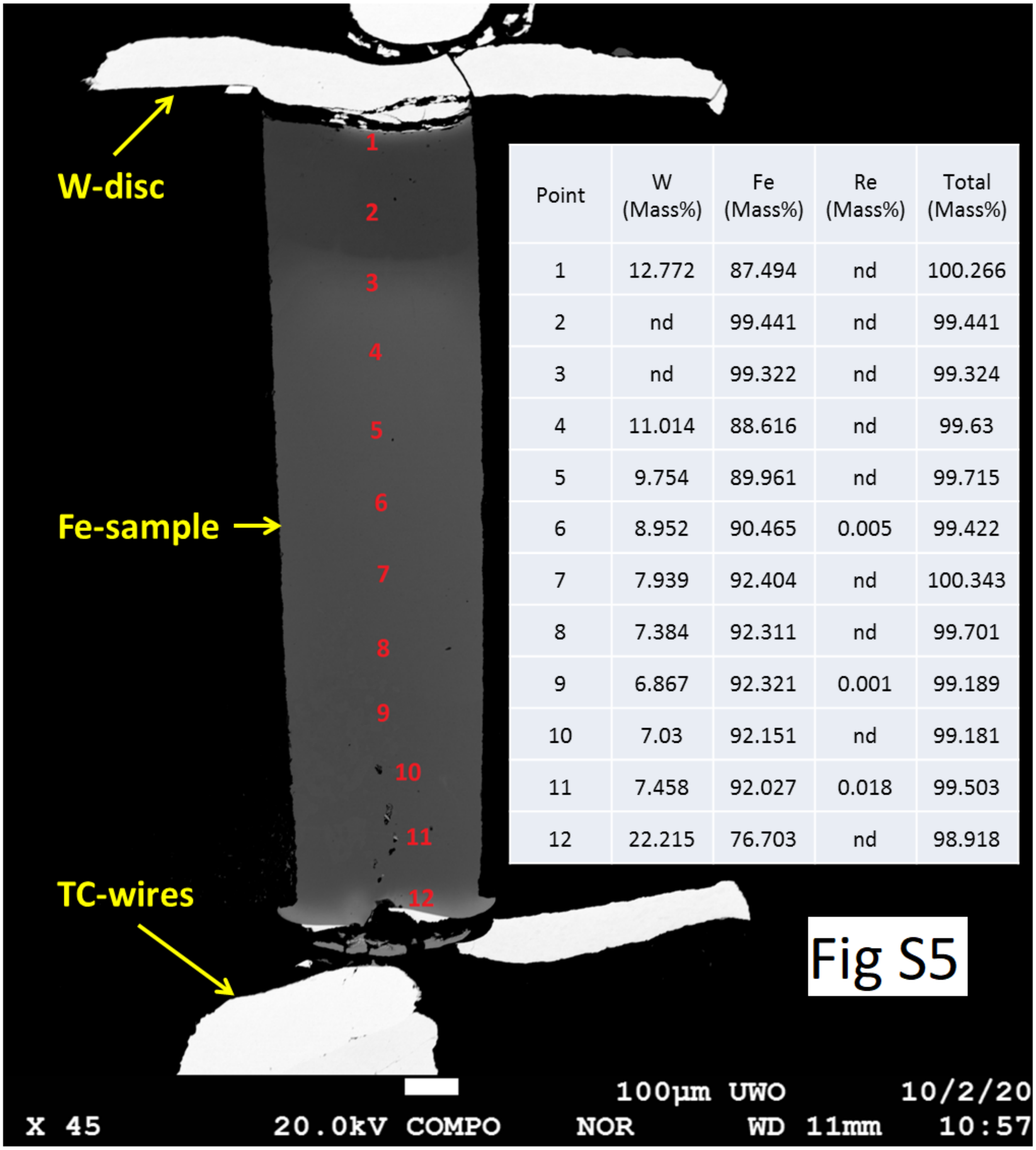


| Point | W (Mass%) | Fe (Mass%) | Re (Mass%) | Total (Mass%) |
|---|---|---|---|---|
| 1 | 12.772 | 87.494 | nd | 100.266 |
| 2 | nd | 99.441 | nd | 99.441 |
| 3 | nd | 99.322 | nd | 99.324 |
| 4 | 11.014 | 88.616 | nd | 99.63 |
| 5 | 9.754 | 89.961 | nd | 99.715 |
| 6 | 8.952 | 90.465 | 0.005 | 99.422 |
| 7 | 7.939 | 92.404 | nd | 100.343 |
| 8 | 7.384 | 92.311 | nd | 99.701 |
| 9 | 6.867 | 92.321 | 0.001 | 99.189 |
| 10 | 7.03 | 92.151 | nd | 99.181 |
| 11 | 7.458 | 92.027 | 0.018 | 99.503 |
| 12 | 22.215 | 76.703 | nd | 98.918 |

Fig S5

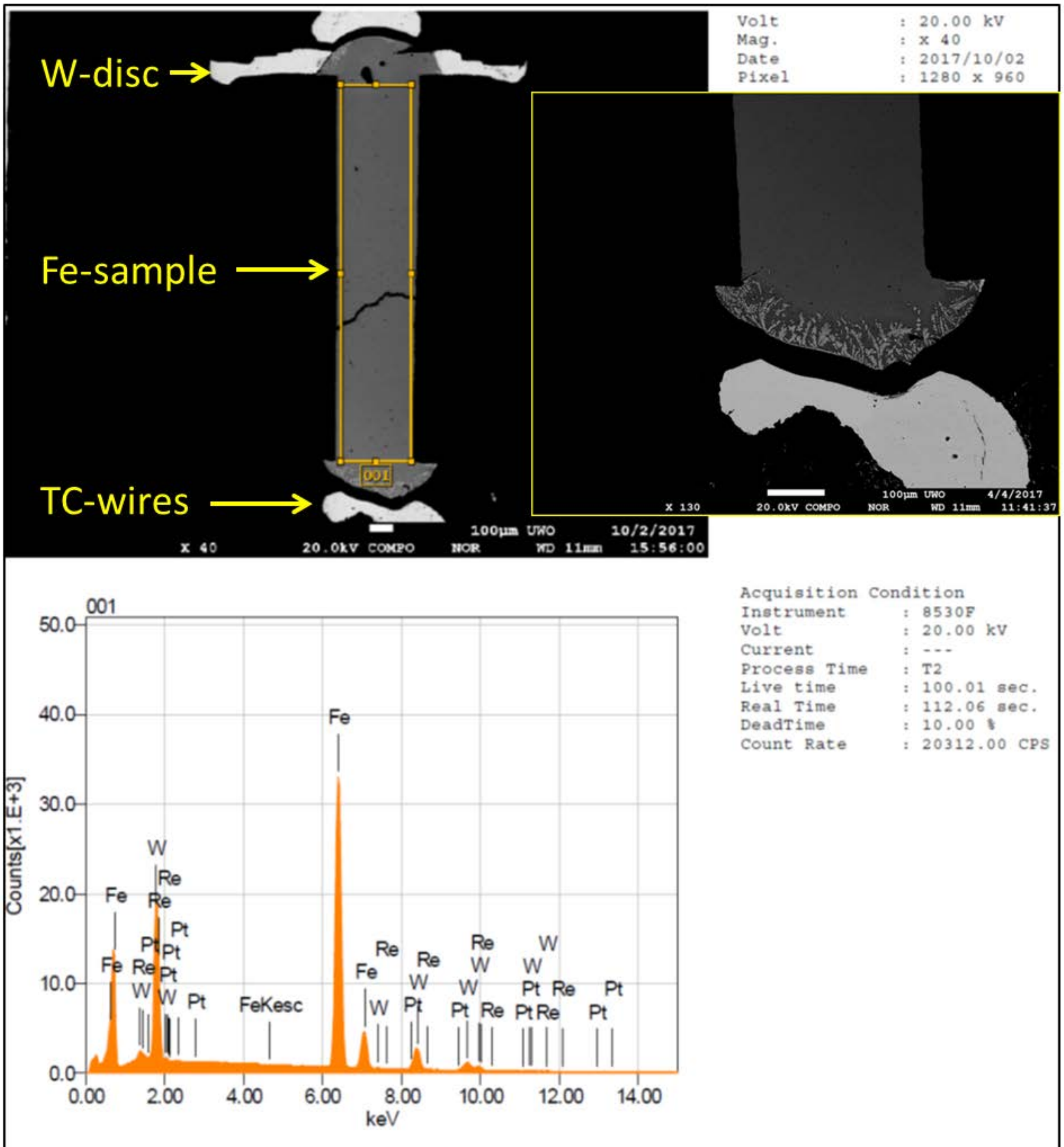


| Formula | mass% | Atom% | Sigma | Net | K ratio | Line |
|---|---|---|---|---|---|---|
| Fe | 68.35 | 87.68 | 0.01 | 18507059 | 38.2233380 | K |
| W | 29.47 | 11.49 | 0.02 | 7315369 | 10.0645696 | M |
| Re* | 1.91 | 0.74 | 0.02 | 474669 | 0.5848864 | M |
| Pt* | 0.27 | 0.10 | 0.01 | 61401 | 0.0750790 | M |
| Total | 100.00 | 100.00 | | | | |

Fig S6

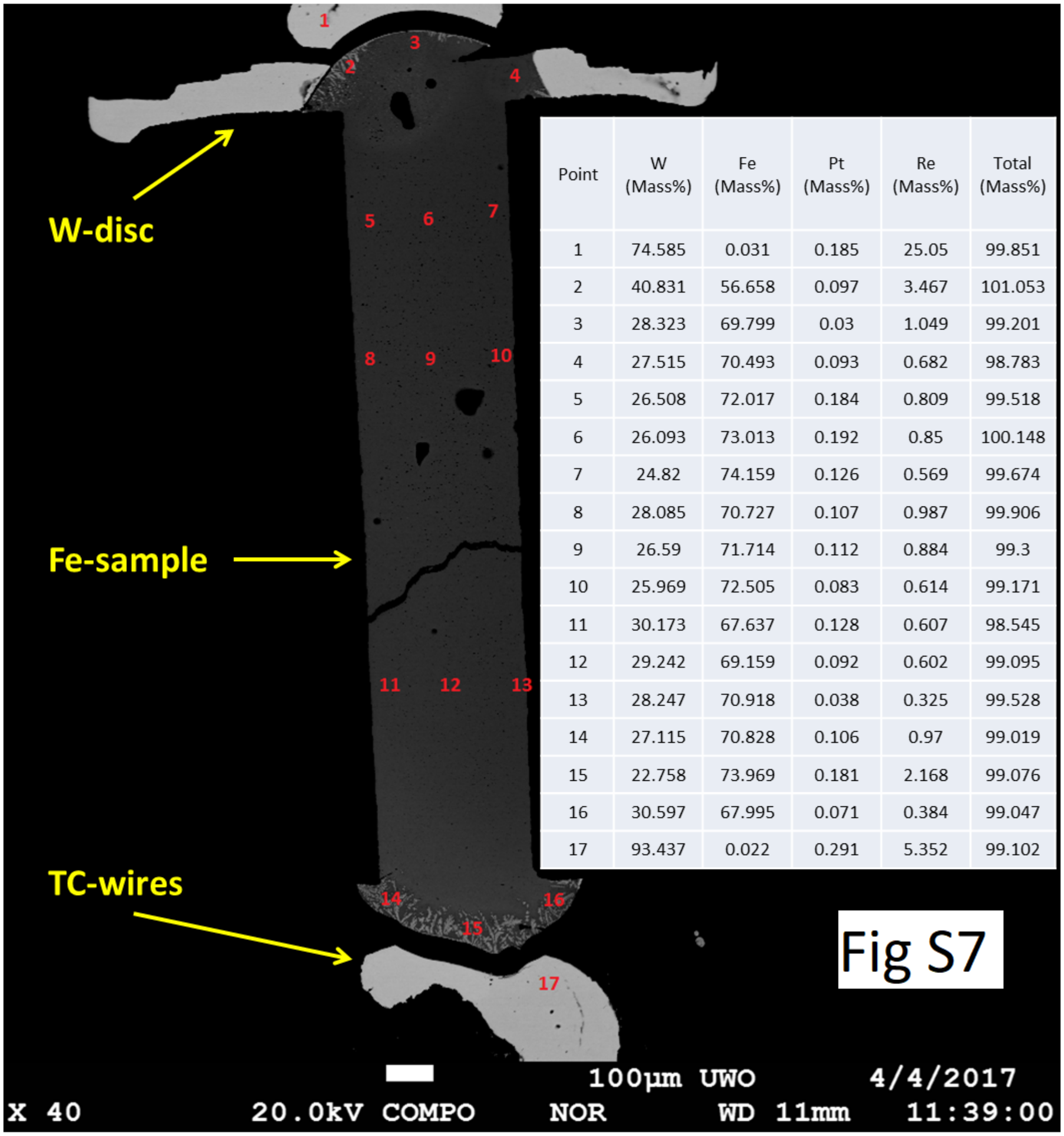


| Point | W (Mass%) | Fe (Mass%) | Pt (Mass%) | Re (Mass%) | Total (Mass%) |
|---|---|---|---|---|---|
| 1 | 74.585 | 0.031 | 0.185 | 25.05 | 99.851 |
| 2 | 40.831 | 56.658 | 0.097 | 3.467 | 101.053 |
| 3 | 28.323 | 69.799 | 0.03 | 1.049 | 99.201 |
| 4 | 27.515 | 70.493 | 0.093 | 0.682 | 98.783 |
| 5 | 26.508 | 72.017 | 0.184 | 0.809 | 99.518 |
| 6 | 26.093 | 73.013 | 0.192 | 0.85 | 100.148 |
| 7 | 24.82 | 74.159 | 0.126 | 0.569 | 99.674 |
| 8 | 28.085 | 70.727 | 0.107 | 0.987 | 99.906 |
| 9 | 26.59 | 71.714 | 0.112 | 0.884 | 99.3 |
| 10 | 25.969 | 72.505 | 0.083 | 0.614 | 99.171 |
| 11 | 30.173 | 67.637 | 0.128 | 0.607 | 98.545 |
| 12 | 29.242 | 69.159 | 0.092 | 0.602 | 99.095 |
| 13 | 28.247 | 70.918 | 0.038 | 0.325 | 99.528 |
| 14 | 27.115 | 70.828 | 0.106 | 0.97 | 99.019 |
| 15 | 22.758 | 73.969 | 0.181 | 2.168 | 99.076 |
| 16 | 30.597 | 67.995 | 0.071 | 0.384 | 99.047 |
| 17 | 93.437 | 0.022 | 0.291 | 5.352 | 99.102 |

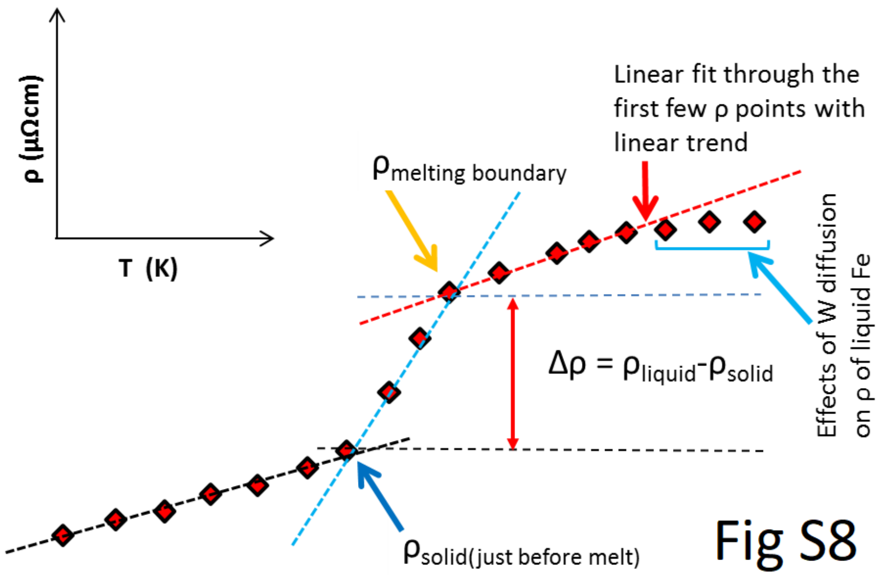
ρ (µΩcm)
T (K)
Linear fit through the first few ρ points with linear trend
ρmelting boundary
Δρ = ρliquid-ρsolid
Effects of W diffusion on ρ of liquid Fe
ρsolid(just before melt)
Fig S8

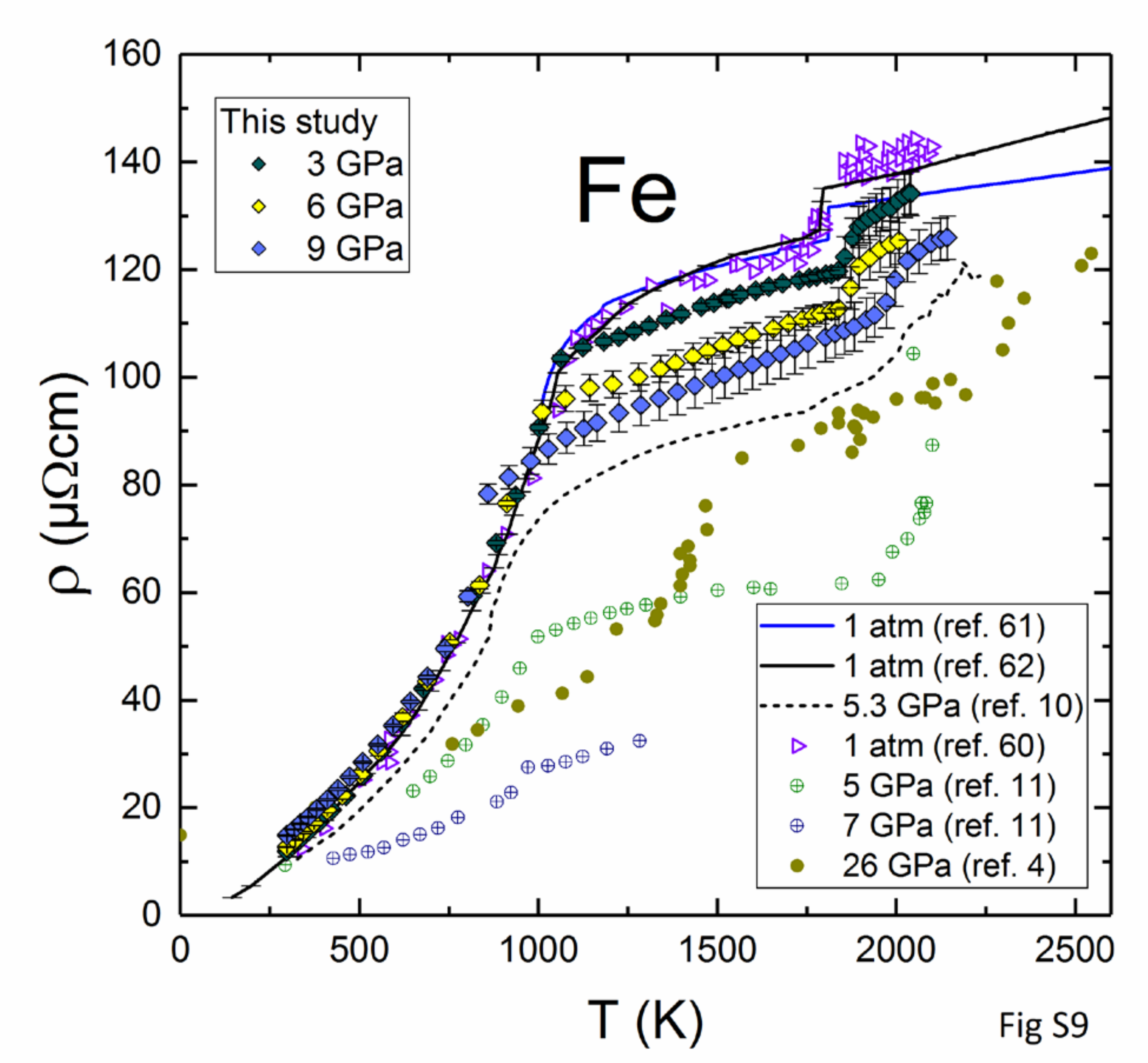
This study
3 GPa
6 GPa
9 GPa
Fe
1 atm (ref. 61)
1 atm (ref. 62)
5.3 GPa (ref. 10)
1 atm (ref. 60)
5 GPa (ref. 11)
7 GPa (ref. 11)
26 GPa (ref. 4)
ρ (μΩcm)
T (K)
0
20
40
60
80
100
120
140
160
0
500
1000
1500
2000
2500

Fig S9

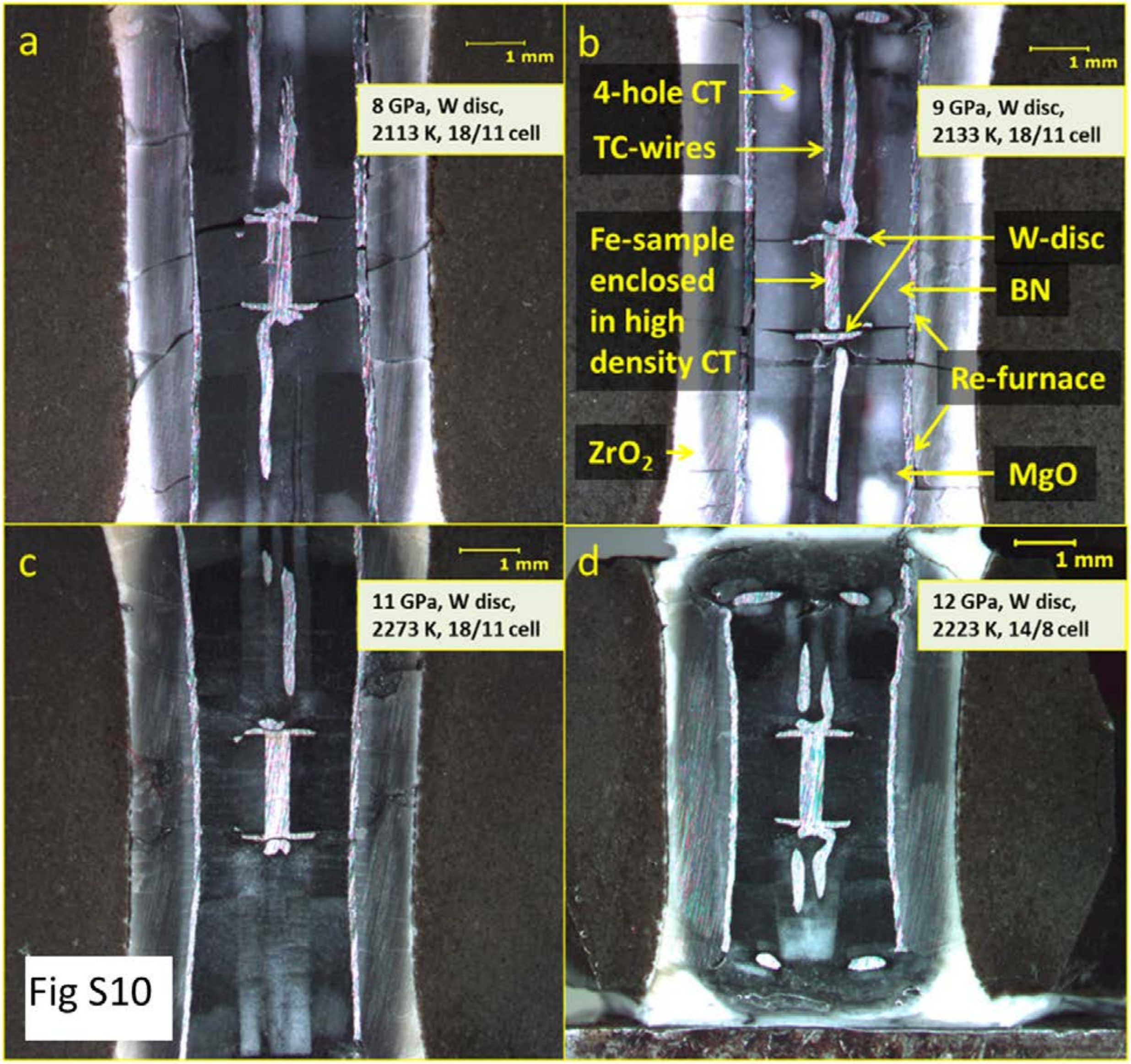
a
1 mm
8 GPa, W disc,
2113 K, 18/11 cell
b
1 mm
4-hole CT
TC-wires
9 GPa, W disc,
2133 K, 18/11 cell
Fe-sample
enclosed
in high
density CT
W-disc
BN
Re-furnace
ZrO2
MgO
c
1 mm
11 GPa, W disc,
2273 K, 18/11 cell
d
1 mm
12 GPa, W disc,
2223 K, 14/8 cell
Fig S10
